# Evaluation of novel derivatisation reagents for the analysis of oxysterols

**DOI:** 10.1016/j.bbrc.2014.01.173

**Published:** 2014-04-11

**Authors:** Peter J. Crick, Jennifer Aponte, T. William Bentley, Ian Matthews, Yuqin Wang, William J. Griffiths

**Affiliations:** a Institute of Mass Spectrometry, College of Medicine, Swansea University, Singleton Park, Swansea SA2 8PP, UK; b College of Engineering, Swansea University, Singleton Park, Swansea SA2 8PP, UK

**Keywords:** 3β-HCA, 3β-hydroxycholest-5-en-(25R)-26-oic acid, 4-DMAP, 4-dimethylaminopyridine, API, atmospheric pressure ionisation, EADSA, enzyme-assisted derivatisation for sterol (or steroid) analysis, EBI2, Epstein–Barr virus induced gene 2, ER, estrogen receptor, ESI, electrospray ionisation, GC, gas chromatography, GP, Girard P, LC, liquid chromatography, LIT, linear ion trap, LXR, liver X receptor, MRM, multiple reaction monitoring, MS, mass spectrometry or mass spectrum, MS*^n^*, MS with multistage fragmentation, RIC, reconstructed ion chromatogram, ROR, retinoic acid related orphan receptor, Girard P reagent, Derivatisation, Liquid chromatography, Mass spectrometry, Sterols

## Abstract

Oxysterols are oxidised forms of cholesterol that are intermediates in the synthesis of bile acids and steroid hormones. They are also ligands to nuclear and G protein-coupled receptors. Analysis of oxysterols in biological systems is challenging due to their low abundance coupled with their lack of a strong chromophore and poor ionisation characteristics in mass spectrometry (MS). We have previously used enzyme-assisted derivatisation for sterol analysis (EADSA) to identify and quantitate oxysterols in biological samples. This technique relies on tagging sterols with the Girard P reagent to introduce a charged quaternary ammonium group. Here, we have compared several modified Girard-like reagents and show that the permanent charge is vital for efficient MS*^n^* fragmentation. However, we find that the reagent can be extended to include sites for potential stable isotope labels without a loss of performance.

## Highlights

• New derivatisation reagents for LC–MS analysis of oxysterols.
• New reagents based on Girard P give high ion-currents and informative LC–MS*^n^* spectra.
• Permanent charge is vital for efficient MS*^n^* fragmentation.
• New reagents offer greater scope for incorporation of isotope labels.

## Graphical abstract

**Figure:**
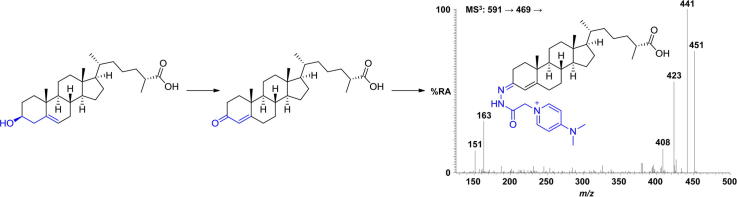

## 1 Introduction

Oxysterols are neutral lipids formed by oxidation of cholesterol either by enzymatic oxidation (especially by enzymes of the cytochrome P450 family) or by autoxidation. As well as acting as key components of the bile acid biosynthesis pathways [1], oxysterols have been shown to bind to liver X receptors (LXRs) [2,3], retinoic acid related orphan receptors (RORs) [4] and the G protein-coupled receptor Epstein–Barr virus induced gene 2 (EBI2) [5,6]. Very recently (25R)26-hydroxycholesterol has been shown to promote proliferation of breast cancer cell lines by binding to the estrogen receptor (ER) α [7,8].

Sterols are typically under-represented in global lipidomics studies which are dominated by charged species (e.g. phospholipids) or those readily ionised due to the presence of a functional group such as an amine (e.g. sphingosine, sphingomyelin). The sterolome itself is dominated by cholesterol which is present at a level three orders of magnitude higher than the most abundant oxysterols. In addition, the lack of a strong chromophore and poor ionisation characteristics in mass spectrometry (MS) make the analysis and accurate quantitation of oxysterols challenging. Gas chromatography (GC)–MS with prior derivatisation to trimethylsilyl ethers is considered the ‘gold standard’ for oxysterol analysis but a number of liquid chromatography (LC)–MS approaches have also been reported (for a recent review see Ref. [9]).

Chemical derivatisation is commonly used in LC–MS methods to improve the ionisation characteristics of the biological molecules of interest [10]. An effective derivatisation reagent should: (1) react in near-quantitative yield with the analyte of interest (e.g. by ‘click’ chemistry [11]); (2) incorporate a permanently charged or readily ionised functional group (e.g. an amine); and preferably (3) be amenable to stable-isotope labelling for use in relative and absolute quantitation experiments.

**Fig. 1:**
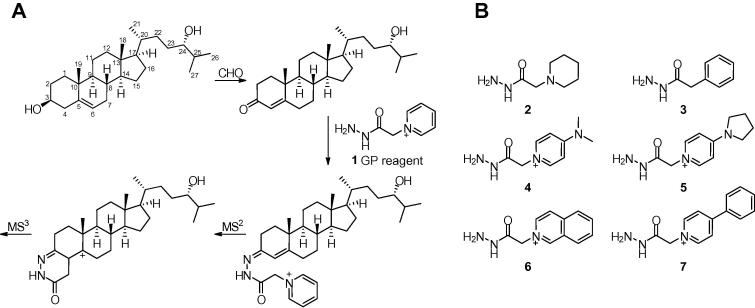
(A) Numbering of the cholesterol backbone and outline of EADSA exemplified with 24S-hydroxycholesterol. (B) Novel derivatisation reagents **2**–**7** used in this study. CHO: cholesterol oxidase; GP: Girard P.

We have developed an LC–electrospray ionisation (ESI)–MS method including enzyme-assisted derivatisation for sterol (or steroid) analysis (EADSA) [12]. Sterols are enzymatically oxidised using bacterial cholesterol oxidase before ‘charge-tagging’ with the Girard P (GP) reagent (Fig. 1A). This incorporates a permanently charged quaternary ammonium group which increases solubility in reversed-phase solvents, improves ionisation by several orders of magnitude, and directs fragmentation of the sterol backbone to provide structurally informative MS*^n^* (MS with multistage fragmentation) spectra.

As part of a project to incorporate isotope labels into the GP reagent (Crick et al., manuscript in preparation [13,14]) we have synthesised and tested a range of charge tags incorporating a variety of functional groups. Here we evaluate the performance of these reagents and discuss their merits and limitations.

## 2 Materials and methods

A list of the sources of reagents and equipment along with a detailed protocol for EADSA is given in Ref. [12].

### 2.1 Solvents and reagents

HPLC grade solvents were from Fisher Scientific (Loughborough, UK). GP reagent (1-(carboxymethyl)pyridinium chloride hydrazide) was from TCI Europe (Oxford, UK) and 2-(1-piperidinyl)acetohydrazide was from Fluorochem (Hadfield, UK). Phenylacetic hydrazide and cholesterol oxidase from *Streptomyces* sp. were from Sigma–Aldrich (Dorset, UK). Certified Sep-Pak tC18 200 mg and Oasis HLB 60 mg cartridges were from Waters (Elstree, UK). Pooled serum from male AB plasma was from Sigma–Aldrich. Chemicals used for the synthesis of derivatisation reagents **4**–**7** were from Sigma–Aldrich.

### 2.2 Synthesis of derivatisation reagents

Charge tags were synthesised using a modification of the procedure reported by Girard and Sandulesco [15]. A solution of heterocycle (4-dimethylaminopyridine, 4-pyrollopyridine, isoquinoline or 4-phenylpyridine, 12.6 mmol) and ethyl bromoacetate (1.4 mL, 12.6 mmol) in absolute ethanol (10 mL) was heated at reflux for 4 h. The mixture was slowly cooled to 0 °C and hydrazine hydrate (788 μL of 78–80% solution in H_2_O, ∼12.6 mmol) was added dropwise. The resulting white precipitate was recovered by filtration and dried under reduced pressure to afford the desired derivatisation reagent (**4**, **5**, **6** or **7**) in 80–95% yield.

### 2.3 Extraction of oxysterols from serum

Serum (100 μL) was added dropwise to a solution of 24(R/S)-[25,26,26,26,27,27,27-^2^H_7_] hydroxycholesterol (20 ng) in absolute ethanol (1.05 mL total volume) in an ultrasound bath. After 5 min, water (350 μL) was added, ultrasonicated for a further 5 min then centrifuged at 14,000*g* for 30 min at 4 °C. A Waters Sep-Pak tC18 cartridge was rinsed with 4 mL of absolute ethanol followed by 6 mL of 70% ethanol and the serum in 70% ethanol (1.5 mL) was applied to the column. The flow-through was collected and combined with a wash of 5.5 mL of 70% ethanol to give SPE-1-Fr-1 containing oxysterols. The cartridge was washed with a further 4 mL of 70% ethanol to give SPE-1-Fr-2, before cholesterol was eluted with 2 mL of absolute ethanol (SPE-1-Fr-3). Finally, the column was washed with 2 mL of absolute ethanol to give SPE-1-Fr-4. The fraction containing oxysterols (SPE-1-Fr-1) was dried under reduced pressure using a vacuum concentrator.

### 2.4 Enzyme-assisted derivatisation

The dried fractions were reconstituted in 100 μL propan-2-ol and 1 mL potassium phosphate buffer (50 mM, pH 7) containing 3 μL of cholesterol oxidase (2 mg/mL H_2_O, 44 U/mg protein) was added. The mixture was incubated at 37 °C for 1 h when the reaction was stopped by addition of methanol (2 mL). Acetic acid (150 μL) was added followed by 0.8 mmol of the derivatisation reagent to be tested. The mixture was vortexed until all of the solid was dissolved then incubated at room temperature overnight in the dark.

### 2.5 Removal of excess reagent

To remove excess reagent while retaining all of the compounds of interest, a recycling procedure was used. The procedure is similar to that previously reported [12] but a Waters Oasis HLB 60 mg cartridge was used in place of the Sep-Pak tC18.

An Oasis HLB cartridge was pre-conditioned with methanol (6 mL), 10% methanol (6 mL) and finally 70% methanol (4 mL). The reaction mixture (in ∼3 mL 70% methanol) was loaded onto the cartridge and the flow-through collected. The column was washed with 70% methanol (1 mL) followed by 35% methanol (1 mL). Water (4 mL) was added to the combined eluent to give ∼9 mL 35% methanol. This solution was applied to the same cartridge and the flow-through collected, along with a wash of 17% methanol (1 mL). Water (9 mL) was added to the eluent to give ∼19 mL 17% methanol. This solution was applied to the column followed by a wash of 10% methanol (6 mL). Finally, the oxysterols were eluted from the cartridge using methanol (3 × 1 mL) then ethanol (1 mL) to give SPE-2-Fr-1, Fr-2, Fr-3 and Fr-4. Aliquots of SPE-2-Fr-1 and Fr-2 were combined and diluted to 60% methanol for analysis by LC–MS.

### 2.6 LC–ESI–MS*^n^* on the LTQ-Orbitrap

Separation of oxysterols was performed on an UltiMate 3000 HPLC system (Dionex) using a Phenomonex Kinetex C18 column (50 × 2.1 mm, 1.7 μm particles). Mobile phase A consisted of 33.3% methanol, 16.7% acetonitrile, 50% water and 0.1% formic acid. Mobile phase B consisted of 63.3% methanol, 31.7% acetonitrile, 5% water and 0.1% formic acid. The flow rate was 200 μL/min. The gradient started at 20% mobile phase B for 1 min before rising to 80% mobile phase B over the next 7 min. After a further 5 min the gradient returned to 20% mobile phase B over 6 s before re-equilibration for 3 min 54 s to give a total run time of 17 min. The eluent was directed to the atmospheric pressure ionisation (API) source of an LTQ-Orbitrap Velos (Thermo Fisher, San Jose, CA, USA). 35 μL of the mixture to be analysed was injected onto the column and a targeted multiple reaction monitoring (MRM)-like method was used to fragment ions of interest in the linear ion trap (LIT), e.g. *m*/*z* 548 → 469→ for 3β-hydroxycholest-5-en-(25R)-26-oic acid (3β-HCA) derivatised with GP reagent. The collision energy was 30 units for the MS^2^ step and 35 units for the MS^3^ step. In addition, a full mass spectrum was recorded in the Orbitrap over the *m*/*z* range 400–610 at 30,000 resolution (FWHM definition).

## 3 Results and discussion

### 3.1 Derivatisation with the GP reagent enhances oxysterol analysis by MS and MS*^n^*

Derivatisation with a reagent possessing an amine moiety is a common technique to enhance the ionisation of analytes of interest in ESI–MS [10]. Primary, secondary and tertiary amines are all readily charged in positive mode ESI to give either protonated or sodiated adducts, while quaternary ammonium species carry a permanent positive charge to typically give a strong response in positive mode ESI–MS. We have previously used the GP reagent **1** to introduce a quaternary ammonium moiety to sterols to enhance detection by LC–ESI–MS (EADSA, Fig. 1A).

**Fig. 2:**
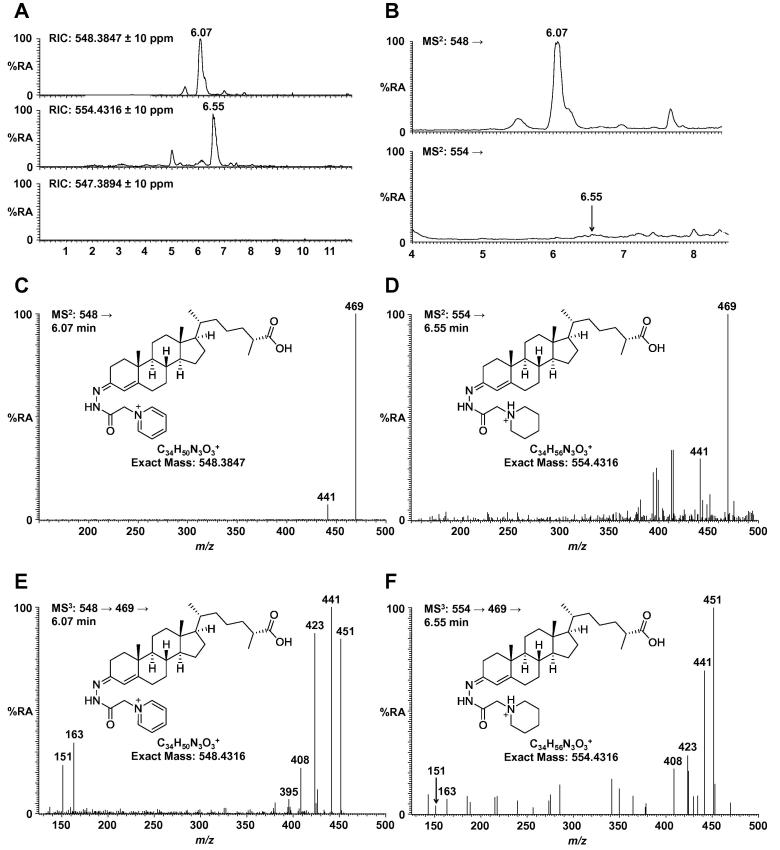
(A) Reconstructed ion chromatograms (RICs) showing 3β-hydroxycholest-5-en-(25R)-26-oic acid (3β-HCA) from a commercial serum sample derivatised with GP reagent ([M]^+^*m*/*z* 548.3847), piperidine reagent **2** ([M+H]^+^*m*/*z* 554.4316) and phenyl reagent **3** ([M+H]^+^*m*/*z* 547.3894). The RICs are plotted on the same *y*-axis. (B) Chromatograms showing MS^2^ transition of 3β-HCA derivatised with GP (*m*/*z* 548→) and piperidine reagent **2** (*m*/*z* 554→). The RICs are plotted on the same *y*-axis. (C) MS^2^ spectrum showing fragmentation of 3β-HCA derivatised with GP reagent (*m*/*z* 548→). (D) MS^2^ spectrum showing fragmentation of 3β-HCA derivatised with piperidine reagent **2** (*m*/*z* 554→). (E) MS^3^ spectrum showing fragmentation of 3β-HCA derivatised with GP reagent (*m*/*z* 548 → 469→). (F) MS^3^ spectrum showing fragmentation of 3β-HCA derivatised with piperidine reagent **2** (554 → 469→).

To assess the importance of the permanent charge we derivatised a commercially available pooled serum sample with reagent **2** (incorporating a piperidine) and reagent **3** (incorporating a phenyl ring) in parallel with the GP reagent (Fig. 1B). Fig. 2A shows reconstructed ion chromatograms (RICs) plotted on the same *y*-axis for 3β-HCA derivatised with reagents **1**–**3**. 3β-HCA is one of the most abundant oxysterols present in healthy human serum at a level of approximately 80 ng/mL and gives a high-intensity peak when derivatised with the GP reagent (Fig. 2A, top panel). We found that derivatisation with piperidine (reagent **2**) gave a peak corresponding to the protonated adduct with an intensity similar to that of the GP reagent (Fig. 2A, middle panel). The sodiated adduct was present at a level of less than 10% of this peak. Therefore, in MS mode, a tertiary amine results in a similar gain in sensitivity as a quaternary ammonium. However, we were unable to identify a peak corresponding to 3β-HCA derivatised with phenyl reagent **3** as either the protonated or sodiated adduct (Fig. 2A, bottom panel). Similar results were obtained for other analytes from the same serum sample.

The sterol backbone is made up almost exclusively of carbon–carbon and carbon–hydrogen bonds plus a carbon–oxygen bond. This makes MS^2^ experiments on underivatised molecules difficult as, in a typical experiment, the fragmentation spectrum is dominated by the non-specific loss of a molecule of water. A key feature of EADSA is a characteristic and predictable loss of pyridine (79 Da) from the GP tag in MS^2^. Importantly, loss of 79 Da is not a commonly observed neutral loss from other biomolecules. In addition, this pathway results in transfer of the positive charge from the derivatising group to the sterol backbone, which can then be fragmented further to afford structurally informative fragments in MS^3^ spectra. Fragmentation of GP-derivatised 3β-HCA results in a major transition of *m*/*z* 548 → 469 corresponding to the loss of pyridine (Fig. 2B, top panel and C). A less intense peak is also observed at *m*/*z* 441 assigned to a loss of pyridine along with a molecule of carbon monoxide. However, fragmentation of 3β-HCA derivatised with piperidine reagent **2** gives no significant peak in the equivalent chromatogram (Fig. 2B, lower panel) although a similar (but weak) fragmentation spectrum can be identified at 6.55 min (Fig. 2D). Further activation of the fragment at *m*/*z* 469 gives an MS^3^ spectrum with peaks corresponding to fragmentation of the sterol A/B-rings (*m*/*z* 151, 163), the residual derivatising group and carboxylate group (*m*/*z* 408, 423, 441, 451). Again, the intensity is considerably higher (thirty fold) for 3β-HCA derivatised with the GP reagent (Fig. 2E) compared with derivatisation using piperidine reagent **2** (Fig. 2F). The weak fragmentation of sterols derivatised with reagent **2** would be of particular importance to techniques that rely exclusively on MS^2^ for identification and quantitation, for example SRM or MRM carried out on a triple-quadrupole mass spectrometer.

### 3.2 The GP reagent can be modified without a loss in performance

Derivatisation reagents may be isotopically labelled to be used in relative and absolute quantification experiments. For example, the TMT and iTRAQ reagents used in proteomics experiments allow the simultaneous quantification of up to 6 samples in a single analytical run [16]. In a similar manner, incorporating stable-isotope labels in the GP reagent will allow the analysis of multiple serum samples in a single LC–ESI–MS injection. However, the relatively small size of the GP reagent limits the number of isotope labels that can be included. If the size of the reagent could be extended without a loss of performance, isotope labels could be incorporated at more positions to increase the utility of this strategy. We have synthesised a library of modified Girard reagents incorporating heterocycles larger than pyridine. For example, 4-dimethylaminopyridine (4-DMAP) can be used to obtain reagent **4** (Fig. 1B) which possesses an additional dimethylamino group. This gives the potential for the introduction of two isotopically coded methyl groups (e.g. CD_3_ in place of CH_3_) thus increasing the scope for multiplexing.

In a similar fashion, incorporation of 4-pyrollopyridine, isoquinoline, or 4-phenylpyridine gives reagents **5**–**7**, again with additional sites for potential isotope labelling (Fig. 1B). All of the syntheses are straightforward and high yielding (typically 80–95%) and can be carried out on large scales (∼10 g). The reagents are obtained as white solids that can be used immediately without the need for time consuming purification steps.

**Fig. 3:**
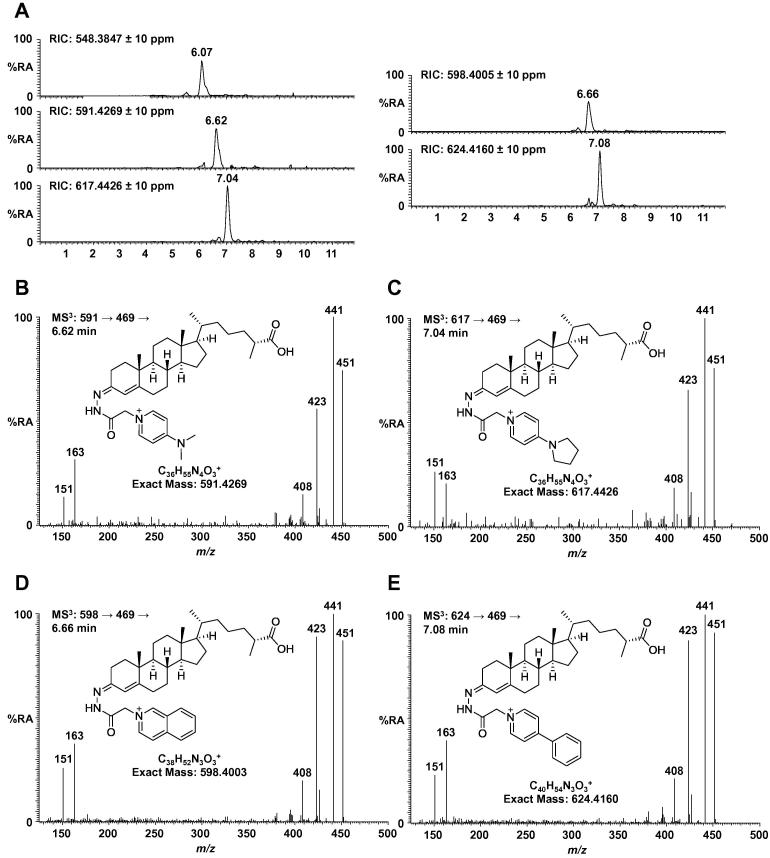
(A) RICs showing 3β-HCA from a commercial serum sample derivatised with GP reagent ([M]^+^*m*/*z* 548.3847), 4-DMAP reagent **4** ([M]^+^*m*/*z* 591.4269), 4-pyrollopyridine reagent **5** ([M]^+^*m*/*z* 617.4426), isoquinoline reagent **6** ([M]^+^*m*/*z* 598.4005) and 4-phenylpyridine reagent **7** ([M]^+^*m*/*z* 624.4160). The RICs are plotted on the same *y*-axis. (B) MS^3^ spectrum showing fragmentation of 3β-HCA derivatised with 4-DMAP reagent **4** (*m*/*z* 591 → 469→). (C) MS^3^ spectrum showing fragmentation of 3β-HCA derivatised with 4-pyrollopyridine reagent **5** (*m*/*z* 617 → 469→). (D) MS^3^ spectrum showing fragmentation of 3β-HCA derivatised with isoquinoline reagent **6** (*m*/*z* 598 → 469→). (E) MS^3^ spectrum showing fragmentation of 3β-HCA derivatised with 4-phenylpyridine reagent **7** (*m*/*z* 624 → 469→).

To compare the performance of reagents **4**–**7** a commercially available pooled serum sample was derivatised with each charge tag along with the GP reagent. As above, we compared the intensities of the peaks corresponding to 3β-HCA. The four new reagents give a similar MS response to that of the GP reagent suggesting that the dominant factor in determining ionisation efficiency is the presence of a quaternary ammonium ion (Fig. 3A, note the chromatograms are plotted on the same *y*-axis). Modifications to the reagent can be used to ‘tune’ the retention time of analytes of interest; for example an additional ring at the 4 position increases the retention time by around 1 min (Fig. 3A, compare top left panel with bottom left and bottom right one). Such modifications could be useful for the analysis of polar molecules that are poorly retained on reversed-phase columns.

Oxysterols derivatised with reagents **4**–**7** fragment in a similar way to those tagged with the GP reagent. In the case of 3β-HCA, neutral loss of the heterocyclic portion of the charge tag gives the same fragment at *m*/*z* 469 which can again be fragmented further to give an MS^3^ spectrum similar to that obtained from the GP reagent (Fig. 3B–E). Incorporation of an amine at the 4 position of the heterocycle (i.e. using 4-DMAP or 4-pyrollopyridine) lowers the efficiency of the MS^3^ fragmentation by a factor of five. However, structurally informative fragments are still observed allowing confident identification of the analytes of interest.

In conclusion we have shown that the GP reagent can be modified without a loss of performance. Replacing the quaternary ammonium moiety with a tertiary amine does not affect ionisation in the ESI–MS mode but greatly reduces the efficiency of MS^2^ fragmentation, thus removing a key benefit of EADSA. However, replacing pyridine with other heterocycles in the GP reagent does not significantly affect the performance of the reagent, giving the potential for future synthesis of multiplexed series of isotopically labelled tags.
